# Progress on the Regulation of Ruminant Milk Fat by Noncoding RNAs and ceRNAs

**DOI:** 10.3389/fgene.2021.733925

**Published:** 2021-11-01

**Authors:** QinYue Lu, Zhi Chen, Dejun Ji, Yongjiang Mao, Qianming Jiang, Zhangping Yang, Juan J. Loor

**Affiliations:** ^1^ College of Animal Science and Technology, Yangzhou University, Yangzhou, China; ^2^ Joint International Research Laboratory of Agriculture and Agri-Product Safety, Ministry of Education, Yangzhou University, Yangzhou, China; ^3^ Mammalian Nutrition Physiology Genomics, Department of Animal Sciences and Division of Nutritional Sciences, University of Illinois, Urbana, IL, United States

**Keywords:** miRNA, lncRNA, circRNA, ceRNA mechanism, ruminants

## Abstract

Milk fat is not only a key factor affecting the quality of fresh milk but also a major target trait forbreeding. The regulation of milk fat involves multiple genes, network regulation and signal transduction. To explore recent discoveries of pathway regulation, we reviewed the published literature with a focus on functional noncoding RNAs and epigenetic regulation in ruminants. Results indicate that miRNAs play key roles in the regulation of milk fat synthesis and catabolism in ruminants. Although few data are available, merging evidence indicates that lncRNAs and circRNAs act on milk fat related genes through indirect action with microRNAs or RNAs in the ceRNA network to elicit positive effects on transcription. Although precise regulatory mechanisms remain unclear, most studies have focused on the regulation of the function of target genes through functional noncoding RNAs. Data to help identify factors that can regulate their own expression and function or to determine whether self-regulation involves positive and/or negative feedback are needed. Despite the growing body of research on the role of functional noncoding RNA in the control of ruminant milk fat, most data are still not translatable for field applications. Overall, the understanding of mechanisms whereby miRNA, lncRNA, circRNA, and ceRNA regulate ruminant milk fat remains an exciting area of research.

## Introduction

The mammary gland is an exocrine organ that provides nutrients and antibodies needed for the growth of the offspring (non-ruminant and ruminant), and experiences periods it continuously of cell proliferation and differentiation after the neonate is weaned (Oftedal, 2002; Macias and Hinck, 2012). Mammary tissue is composed a vast network of acini with mammary epithelial cells arranged tightly around the acini in a monolayer, all of which play a role in milk synthesis. Milk formation in mammary epithelial cells is induced by various nutrients and hormones in the blood through a series of complex biochemical processes (Tucker, 2000; Li and Capuco, 2008; Coussens and Pollard, 2011). Ruminant milk contains a variety of bioactive fatty acids, such as saturated and conjugated linoleic acid. Milk fat is not only a major factor affecting flavor and nutritional value but also a major economic trait considered in ruminant breeding (Haug et al., 2007; Turck, 2013). Studies have shown that different consumer groups have different requirements for the content and ratio of milk fat in the milk products purchased (Achón et al., 2019; Mohan et al., 2021). The process of milk fat synthesis and utilization is regulated not only by multiple genes, but also by a series of regulators, which exert considerable effects. Thus, the study of milk fat synthesis and the regulation of fatty acid composition is an active research area.

**FIGURE 1:**
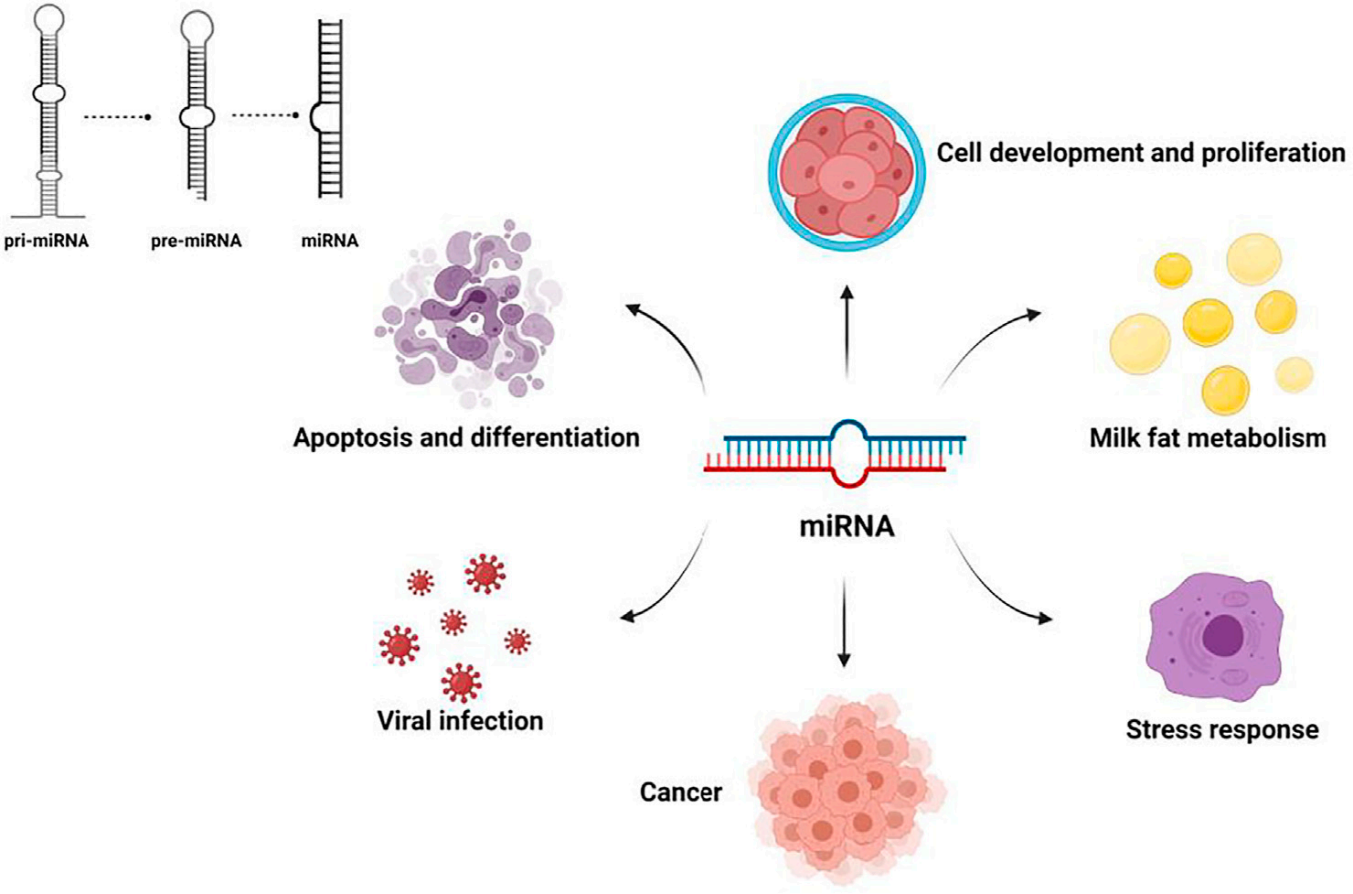
Major functions of microRNAs.

In recent years, an increasing number of studies have shown that noncoding RNAs (ncRNAs), which account for 98% of total RNAs, play important regulatory roles in the process of milk fat synthesis and utilization (Melnik and Schmitz, 2017). For example, by inhibiting target mRNA translation, microRNAs(miRNAs) can regulate the development of tissues and cells, such as mammary, skeletal muscle, and follicles (Figure 1). Long non-coding RNA (lncRNAs) play a key role in development and cancer, and circular RNAs(circRNAs) may be participation coding in biological processes. With the proposal of competitive endogenous RNA mechanisms (ceRNA), researchers have gained a new perspective on transcriptome research. Compared with the miRNA regulatory network, the ceRNA regulatory network is more refined and complex and involves more RNA molecules. Thus, comprehensive studies of ceRNA could help explain some biological phenomena.

The advent of molecular tools has allowed appreciate for the complexity of molecular mechanisms underpinning the regulation of fatty acid metabolism in the mammary gland. It is now recognized that those mechanisms are far more complex than previously known, and a considerable number of regulators need to be mined and identified. In this paper, we focus on mechanisms related to miRNAs, lncRNAs, circRNAs, and ceRNAs in ruminants, research methods and the progress made in recent years in correlating these noncoding RNAs to milk lipid traits. It is believed that this endeavor could help elucidate mechanisms and provide experimental ideas and a theoretical basis for the production of high-quality milk.

## MicroRNAs

A microRNA (miRNA) is an endogenous short noncoding RNA with a length of 18–25 nt (Mattick, 2009). It regulates gene expression at the transcriptional level by inducing degradation of mRNA or blocking its translation.

### Mechanism of miRNA Action

**FIGURE 2:**
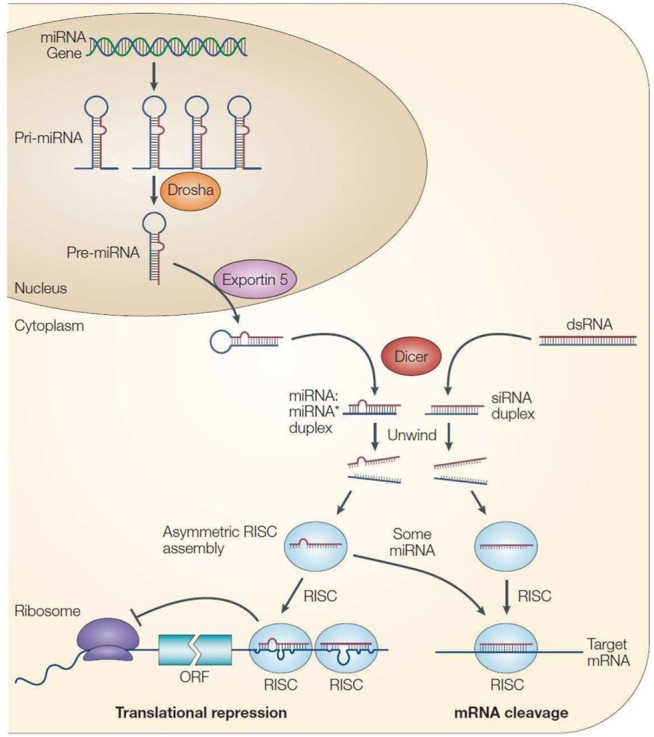
Processing process and mechanism of action of miRNA (He and Hannon, 2004).

Due to the different degrees of complementary pairing of miRNAs with target mRNA sequences in RNA-induced silencing complex (RISC), the way mRNAs regulates target genes also differs (Auyeung et al., 2013). Because the sequence can be completely complementary, almost completely complementary or incompletely complementary, two mechanisms regulate mRNA function. The first method causes the target mRNA to be cut and degraded directly, while the second method inhibits the translation of mRNA (Gäken et al., 2012). Generally, miRNAs are less complementary to target mRNAs in animals, and their mode of action is mainly to inhibit the translation of target mRNA (Figure 2).

The relationship between miRNAs and target genes is not a “one-to-one” correspondence but a “many-to-many” relationship. This means that one miRNA can regulate the expression of multiple target genes, and a target gene may also be jointly regulated by multiple miRNAs (Carthew and Sontheimer, 2009). Various miRNAs and target genes constitute a large gene regulatory network, and this mechanistic diversity makes the process of miRNA regulation of gene expression very complex.

### Methods Used to Research miRNAs

#### Identification of miRNAs and Target Genes

There are three main methods for miRNA identification: direct cloning, bioinformatics prediction, and high-through put sequencing. Among these methods, bioinformatics prediction is currently widely applied in this field. Since miRNA precursors have a typical stem-loop structure and because the inherent characteristics of the miRNA structure make them thermodynamically stable, software can be used with this information to predict miRNAs. High-throughput sequencing can directly perform deep sequencing of miRNAs in cells or tissues at the molecular level and analyze results without referring to the genome sequence. Because of its high sensitivity, large sequencing throughput and low cost, high-throughput sequencing has been widely used across various species (Aldridge and Hadfield, 2012).

#### miRNA Expression Level Detection

Since the length of mature miRNA is only 19–23 nt, its sequence must be extended during reverse transcription before PCR amplification can be carried out. Commonly used methods include the stem-loop method and tailing method. The stem-loop method is based on the use specific stem-loop primers for reverse transcription (Kramer, 2011). The specific stem-loop primers include a universal stem-loop sequence and a 6–8 base reverse complementary to the 3′ end of a mature miRNA. After obtaining cDNA, specific upstream primers and universal primers are used. The downstream primers are used for PCR. The primers can be used for both PCR and RT-qPCR. The tailing method adds a poly(A) tail to the 3′ end of a miRNA and then the primer with a poly(T) and an adapter (adapters) is used to proceed. Reverse transcription is followed by RT-qPCR amplification with specific upstream primers and the universal downstream primers that are included in the kit (Chang et al., 2014).

Methods to detect miRNA expression levels mainly include miRNA gene chip analysis, northern blot analysis and real-time fluorescent quantitative PCR (RT-qPCR) detection (Mendes et al., 2009). Among these methods, a gene chip is mainly used for preliminary detection of miRNA expression patterns. However, detection results may include false positives, which can be further verified by more accurate methods such as Northern blotting and RT-qPCR. Northern blot analysis can detect the content and molecular weight of miRNA, but the sensitivity is low.

#### miRNA Overexpression/Suppression

There are several ways to inhibit miRNA expression.

Gene-editing tools can be used at the gene level. These tools include CRISPR/Cas9 to knock out Dicer or AGO, each of which plays an important role in the miRNA biosynthetic pathway. The use of gene-editing tools results in the loss of all mature miRNAs in the individual sample (Mali et al., 2013). At the nongene level, injection of miRNA analogs (mimics) or agonists (agomirs) is the most commonly used experimental method. The effects of mimics and agomirs are the same as those of mature miRNAs, and both can upregulate the content of corresponding miRNAs in cells. As miRNAs have become a new hot spot in the field of life sciences, these methods have also become powerful tools for miRNA research. In general, overexpression of miRNAs can further reveal miRNA functions.

### Regulation of Ruminant Milk Lipid Synthesis and Utilization by miRNAs

An increasing number of studies have shown that miRNAs are involved in the regulation of fatty acid composition and adipocyte differentiation (Chen et al., 2013; Zhang et al., 2015). One of the main products of ruminants is milk, which has not only nutritional value but also good preventive and health care functions in various diseases (Gil and Ortega, 2019). These functions are closely linked to the composition and content of fatty acids in milk (Parodi, 1997; Gebauer et al., 2011; Kuhnt et al., 2016). Thus, in-depth study and application of regulating unsaturated fatty acids in mammary glands can provide a reference for improving the dietary pattern and approach to raising ruminants.

The principal components of ruminant milk fat are triglycerides (TAG), glycerides and fatty acids. Triglycerides account for a large proportion of these components, highlighting that triglycerides play key roles in determining lipid content of dairy products. There have been a number of studies showing that miRNAs are related to the production of milk TAG in ruminants. For example, Zhang et al. (2019) found that miR-454 and peroxisome proliferator-activated receptor-gamma (PPAR-γ) are directly targeted, and in a previous study PPAR-γ was demonstrated to be involved in milk fat synthesis in bovine mammary epithelial cells (Kadegowda et al., 2009). It has been reported that miR-130a can reduce triglyceride synthesis by regulating PPAR-γ; thus, Yang et al. (2020) reasoned that miR-454 may affect TAG levels in bovine mammary epithelial cells by targeting PPAR-γ, and this conjecture was tested through a series of experiments, which indicated that miR-454 can be utilized to improve the quality of dairy products. Similarly, Chu et al. (2018) found that miR-15b can target the fatty acid synthase gene (FASN), and it had been previously shown that FASN can be directly targeted by miR-24 to regulate TAG concentration and unsaturated fatty acid content in goat mammary epithelial cells; therefore, Wang et al. (2015) validated this these findings with goat mammary epithelial cells and found that miR-15b can regulate TAG content in adipocytes, confirming the key role of this miRNA. Unexpectedly, this experiment also reported that the steroid hormones estradiol and progesterone reduced the expression of miR-15b, but no follow-up study was conducted based on this finding. Whether this reduction in miR-15b can reveal a mechanism of action for some miRNAs requires further investigation.

Numerous similar studies on the regulation of TAG content in ruminant mammary epithelial cells by miRNAs have been performed (Table 1). Overall, most of the relevant research have been based on conjecture and validated based on previous knowledge of the targeted gene loci, and no new targeted gene loci have been demonstrated to be subject to miRNA feedback regulation or to have an effect on TAG content. More in-depth studies of target gene loci can be performed after sufficient knowledge of miRNAs has been obtained.

**TABLE 1:** miRNAs involved in the regulation of TAG content in ruminant milk fat metabolism.

miRNA	Species	Target genes	Functions	References
miR-106b	Bovine	ABCA1	Results in decreased TAG content	Chen et al. (2019a)
miR-29s	Bovine	PPAR-γ	Results in decreased TAG content	Bian et al. (2015)
miR-181b	Goat	INS2	Results in decreased TAG content	Chen et al. (2016a)
miR-152	Bovine	UCP3	Promotes the synthesis of TAG	Shen et al. (2019a)
miR-124a	Bovine	PECR	Promotes the synthesis of TAG	Shen et al. (2019b)
miR-21–3p	Bovine	ELOVL5	Promotes the synthesis of TAG	Li et al. (2019)
miR-142–5p	Goat	CTNNB1	Promotes the synthesis of TAG	Mobuchon et al. (2017)
miR-15b	Goat	FASN	Promotes the synthesis of TAG	Chu et al. (2018)
miR-24	Goat	FASN	Promotes the synthesis of TAG	Wang et al. (2015)
miR-17–5p	Goat	PGC1α	Promotes the synthesis of TAG	Chen et al. (2017)
miR-148a	Goat	PPARα	Promotes the synthesis of TAG	Chen et al. (2017)

Lipid droplets are the main storage sites for intracellular neutral lipids, and newly generated TAGs in ruminant mammary epithelial cells form lipid droplets within lobules of mammary cells. A number of studies have shown that lipid droplets play significant roles in lipid metabolism and storage, membrane transport, protein degradation, and signaling processes. Thus, it is highly desirable to study lipid droplets in ruminant mammary epithelial cells. By examining and contrasting the proportion and content of fat in goat mammary epithelial cells (GMECs) at peak and late lactation,Chen et al. (2018a) found that the expression of miR-183 changed significantly across different lactation periods; thus, they studied the effects of these changes and found that miR-183 plays an important role in the formation of lipid droplets, and further exploration revealed that miR-183 inhibited the accumulation of lipid droplets in GMECs by targeting the MST1 gene, which resulted in a decrease in the TAG concentration.

Tang et al. (2017) investigated the function of miR-27a in BMECs on the basis of previous studies and found that it inhibited lipid droplet accumulation by regulating PPAR-γ. Interestingly, miR-27a was also shown to affect milk fat by regulating PPAR-γ in sheep mammary epithelial cells (Lin et al., 2013), but its function led to the direct reduction in the expression of mRNAs of genes related to TAG synthesis and did not produce direct effects on lipid droplets. There are numerous studies on the relationship between miRNAs and lipid droplet formation in ruminant mammary glands (Table 2). Unfortunately, although previous studies have confirmed that adipose differentiation-related protein (ADFP) and xanthine dehydrogenase (XDH) play indispensable roles in the process of lipid droplet formation and secretion, no relevant studies on the relationship between miRNAs and these two genes in ruminants have been performed to date. It is suggested that subsequent studies be focused on screening miRNAs for ADFP and XDH to further improve the beneficial components in milk.

**TABLE 2:** miRNAs involved in the regulation of lipid droplet synthesis and accumulation in ruminant.

miRNA	Species	Target genes	Functions	References
miR-454	Bovine	PPAR-γ	Inhibition of lipid droplet accumulation	Zhang et al. (2019)
miR-34b	Bovine	DCP1A	Inhibition of lipid droplet accumulation	Wang et al. (2019)
miR-181a	Bovine	ACSL1	Inhibition of lipid droplet synthesis	Lian et al. (2016)
miR-27a	Bovine	PPAR-γ	Inhibition of lipid droplet accumulation	Tang et al. (2017)
miR-130b	Goat	PGC1α	Inhibition of lipid droplet accumulation	Chen et al. (2015)
miR-183	Goat	MST1	Inhibition of lipid droplet accumulation	Chen et al. (2018a)
miR-25	Goat	PGC1β	Inhibition of lipid droplet accumulation	Ma et al. (2018)

Cholesterol is an essential nutrient for the human body, but the long-term intake of large amounts of cholesterol is not conducive to physical health and increases the risk of cardiovascular disease. Cholesterol is widely present in ruminant dairy products, and the study of cholesterol can effectively enhance the nutritional value of dairy products.Chen et al. (2016b) screened miR-15a and miR-30e-5p in a study on the Hippo and Wnt pathways. Through a series of experiments, they demonstrated that miR-15a and miR-30e-5p target YAP1 and LRP6, respectively, and promote cholesterol synthesis in GMECs. Interestingly, a potential feedback relationship between miR-15a and miR-30e-5p was found through these experiments; specifically, miR-30e-5p was shown to inhibit the YAP1 gene by regulating miR-15a. These results suggested that miRNAs do not solely function by regulating genes, and the mechanistic network map of miRNAs must be more complex than is currently understood. LATS1 is a major member of the Hippo pathway, and although previous studies showed that its main function is to participate in myocardial development (Mo et al., 2014), more recent studies have indicated that LATS1 is also associated with milk fat in ruminants. MiR-16a can reduce cholesterol content in BMECs by targeting LATS1, which in turn reduces TAG content (Chen et al., 2019b). Overall, miRNAs regulate cholesterol content in ruminant milk by increasing/decreasing the expression of corresponding metabolic marker genes.

In ruminant mammary lipid metabolism, miRNAs are also involved in the regulation of other processes (Table 3). For example, during lactation, ruminant microvascular endothelial cells secrete large amounts of milk proteins, especially casein (the protein that determines milk specificity), and miRNAs can regulate casein secretion by targeting gene loci. In summary, researchers have screened many different miRNAs related to milk fat across different stages of lactation in ruminants, and most of the miRNAs were found to play regulatory roles in this process. However, compared with other species, some miRNAs and target gene loci in ruminants have not been studied or their functions have not been fully explored. Thus, further studies are required. In addition, the regulatory networks of some miRNAs that have emerged in studies are not perfect. Thus, we recommend that more-intensive studies on these findings be performed to improve the regulatory networks of miRNAs involved in milk fat synthesis and utilization to generate a more precise theoretical basis for improvement of milk quality.

**TABLE 3:** MiRNAs regulate other processes in mammary lipid metabolism in ruminants.

miRNA	Species	Target genes	Functions	References
miR-139	Bovine	GHR, IGF1R	Inhibition of ß-casein synthesis and proliferation	Cui et al. (2017)
chi-miR-8516	Goat	STC1, MMP1	Inhibition of ß-casein synthesis and proliferation	Zhang et al. (2021)
chi-miR-3031	Goat	IGFBP5	Increased expression of ß-casein	Chen et al. (2018b)
miR-135a	Goat	PPLR	Regulation of prolactin secretion	Ji et al. (2015)
miR-99a-3p	Goat	ATP2B1	Promotes milk calcium levels	Chen et al. (2020a)
novel-miR-3880	Goat	ELF2	Reduces ß-casein expression and increases κ-casein secretion	Zhang et al. (2020)
miR-24-3p	Bovine	MEN1	Regulates the synthesis of milk proteins	Qiaoqiao et al. (2019)
miR-486	Bovine	PTEN	Regulation of Phosphoacyl Alcohol Signal Transduction	Li et al. (2015a)
miR-103	Bovine	PKAN3	Accelerate *de novo* synthesis of fatty acids	Cai et al. (2017)
miR-224	Bovine	ACADM	Increased apoptosis rate	Shen et al. (2017)

## Long Non-Coding RNAs

LncRNAs constitute a class of RNA and are >200 nt in length. They do not have the ability to encode proteins and are widespread in the cytoplasm and nucleus of ruminants. Studies have shown that lncRNAs play key roles in many important biological processes, including transcription, splicing, translation, protein localization, cellular structural integrity, genomic imprinting, the cell cycle, apoptosis, and the heat shock response.

### Mechanism of Action of LncRNAs

**FIGURE 3:**
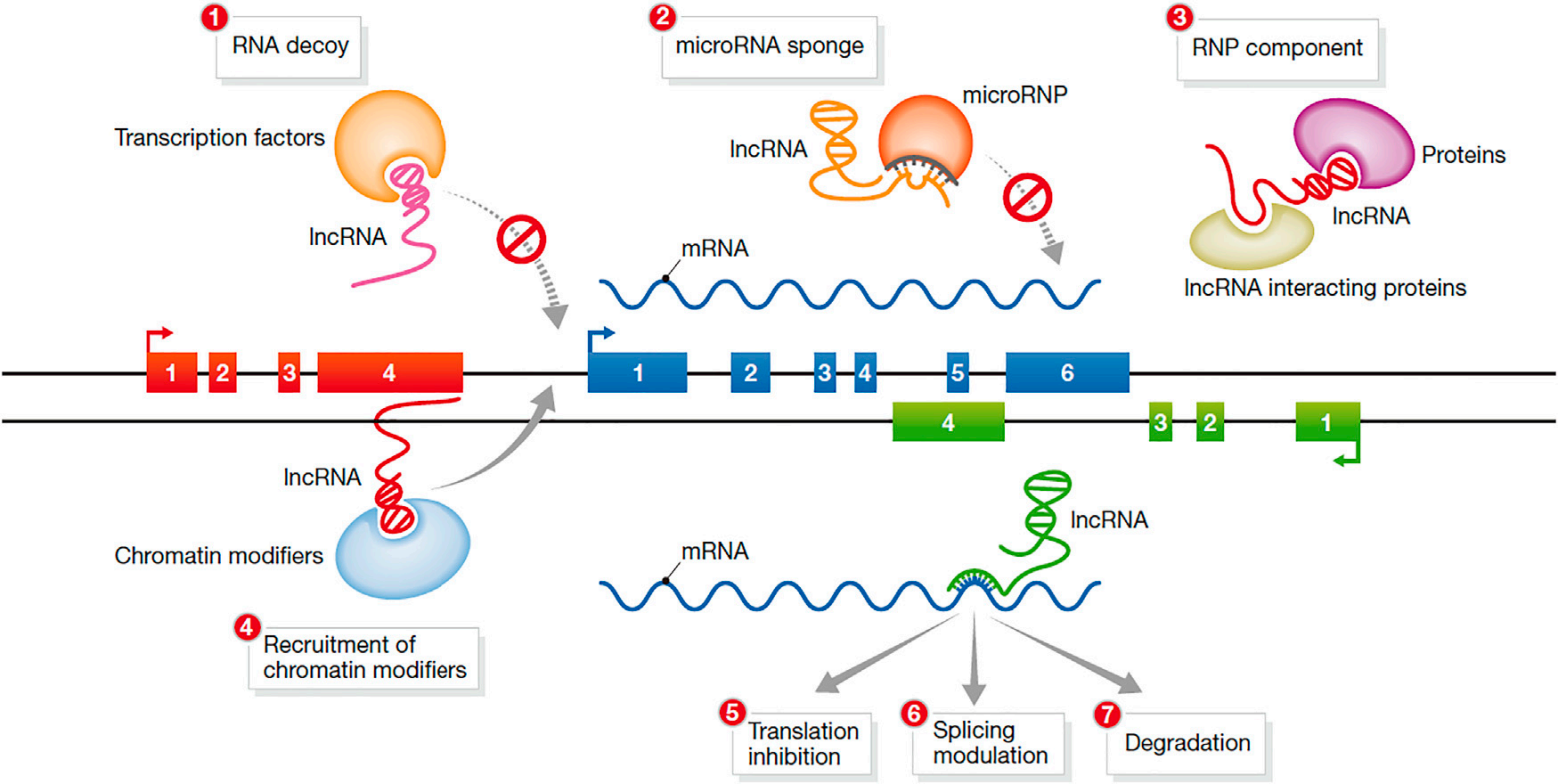
Mechanisms of action of lncRNAs (Hu et al., 2012).

The mechanism of action of lncRNAs is complex and not yet fully understood. According to recent research, the mechanism of lncRNAs has five main aspects (Figure 3).

In the first mechanism, lncRNAs function as a signal molecule. Studies have shown that under different stimulus conditions and signaling pathways, some lncRNAs are specifically transcribed and act as signal transduction molecules to participate in the induction of specific signaling pathways. For example, in the process of the DNA damage response, the expression of many lncRNAs is induced. Thus, lncRNAs are signaling molecules that respond to different stimuli (Noh et al., 2018)

In the second mechanism, lncRNAs act as bait molecules. Some lncRNAs bind to DNA and proteins after transcription, inhibit the function of transcription factors and block the transcription of target genes. Thus, they can block the function of the molecule and the signaling pathway (Chen, 2016).

In the third mechanism, lncRNAs act as scaffold molecules between proteins. LncRNAs can recruit a variety of proteins to form ribonucleoprotein complexes; thus, multiple related transcription factors can bind to the lncRNA molecule (Rinn and Chang, 2012). After multiple signaling pathways are activated, the information can be integrated through the same lncRNA to achieve signal transmission between different signaling pathways.

In the fourth mechanism, lncRNAs acts as guide molecules. LncRNAs can facilitate the binding or removal of nuclear proteins, chromatin-modifying enzymes and other regulatory factors at specific sites of action, thereby regulating the transcription of downstream molecules. This mode of regulation can be realized through either cis-regulation or trans-regulation.

In the fifth mechanism, lncRNAs encode functional small peptides. Although lncRNAs cannot encode proteins, they have the ability to encode functional small peptides and perform biological functions through the small peptides they encode.

### LncRNA Research Methods

To reveal the molecular mechanisms of lncRNAs, a variety of molecular biological techniques need to be used. Currently, a number of established technologies are applied in lncRNA research, including microarrays, RNA-seq, Northern blotting, real-time quantitative reverse transcription-polymerase chain reaction (qRT-PCR), fluorescence *in situ* hybridization (FISH), RNA interference (RNAi), and RNA-binding protein immunoprecipitation (RIP).

Microarrays and RNA-seq are effective tools for high-throughput detection of lncRNA expression. Ørom et al. (2010) used a variety of human cell lines to perform lncRNA expression profiling and detected 3,019 lncRNAs with an average length of 800 nucleotides.

Northern blotting and qRT-PCR are not only widely applied to analyze expression level of lncRNAs but are also often used to verify the authenticity of microarray experimental results. Chang et al. (2018) used qRT-PCR and Northern blotting to detect the expression of the lncRNA HOTAIR and ultimately found that HOTAIR has an anticancer effect by promoting the simultaneous expression of CCND1 or CCND2 in ovarian cancer.

Similar to Northern blotting, FISH also uses the principle of molecular hybridization. Some researchers use this technology to detect and locate specific lncRNAs in cells, reflecting the greatest advantage of FISH, which can be used for cell localization research. Huang et al. (2017) used this method to identify the relationship between the expression of the lncRNA AK023391 and the clinicopathological characteristics and prognosis of gastric cancer patients and ultimately showed that the lncRNA AK023391 may be a potential biomarker for gastric cancer.

The above mentioned techniques are mainly used for the qualitative and quantitative analysis of lncRNAs, but in research on lncRNA function, RNAi and RIP are more commonly used tools. RNAi is widely used to silence specific lncRNAs. For example, Liu et al. (2019) used this technology to study why lncRNA-MEG3 significantly upregulates myosin heavy chain and ultimately proved that the lncRNA-MEG3 promotes the differentiation of bovine skeletal muscle by interacting with miRNA-135 and MEF2C.

RIP is another technology used for studying interactions between RNAs and proteins, and it can play a role in screening relevant proteins that bind to lncRNAs. Recently, RIP technology has been used to make progress in understanding RNA-protein interactions. The combination of RIP and microarrays was developed into the RIP-Chip, and the combination of RIP and RNA-seq was developed into RIP-seq.

### Regulation of Ruminant Milk Lipids by lncRNA

Through in-depth research, it was confirmed that on lncRNAs play important roles in cell differentiation, epigenetics, cell cycle regulation, and the occurrence and development processes of many diseases.

Through high-throughput sequencing or chip technology, it has been confirmed that many lncRNAs are related to the production, development, metabolic regulation and fatty acid composition of adipose tissue and play important roles in the process of fat accretion. For example, Ji et al. (2020) performed a genome-wide analysis of lncRNAs in the GMECs of Laoshan dairy goats at different lactation periods using methods such as GO annotation and KEGG analysis. Similarly, Yang et al. (2018) carried out related work on the Chinese Holstein mammary gland. The findings of these studies have been instrumental in the subsequent in-depth study of the regulatory mechanisms of mammary gland development and lactation in ruminants.

The lncRNA expression profile related to milk fat in ruminants has gradually improved. However, subsequent in-depth exploration of the regulatory mechanisms controlling milk fat has rarely been reported to date, and most conclusions offered are based solely on speculation. For example, on the basis of sequencing results (Yang et al., 2018), TCONS00075230 was inferred to possibly be involved in regulating the proliferation of mammary epithelial cells by interacting with the IL1B gene in the mammary gland of dairy cows. And another experiment performed simultaneously revealed that the expression of four lncRNAs (TCONS00040268, TCONS00137654, TCONS00071659, and TCONS00000352) decreased with the overexpression of miR-221, which was also confirmed to regulate the proliferation of BMECs by targeting genes in later studies (Jiao et al., 2019). Whether these four lncRNAs have an effect on milk fat metabolism in cattle through miR-221 is a question worth studying.

Overall, most of the current studies on lncRNAs in ruminant milk fat relied on bioinformatics predictions, and only a few studies have explored the mechanism of action at the mammary gland level. However, lncRNAs have been confirmed to have an important role in control of milk fat. Of interest, some studies on lncRNAs in the mammary gland have been performed in humans (Sandhu et al., 2016), and whether the study of ruminant mammary development can be used for reference is worth considering.

## Circular RNAs

### Mechanisms of Action of circRNAs

The mechanisms by which circRNAs functions mainly includes acting as a competitive endogenous RNA, regulating alternative splicing or translation, regulating expression of parental genes, and performing biological functions by interacting with proteins.

#### As Competitive Endogenous RNAs

**FIGURE 4:**
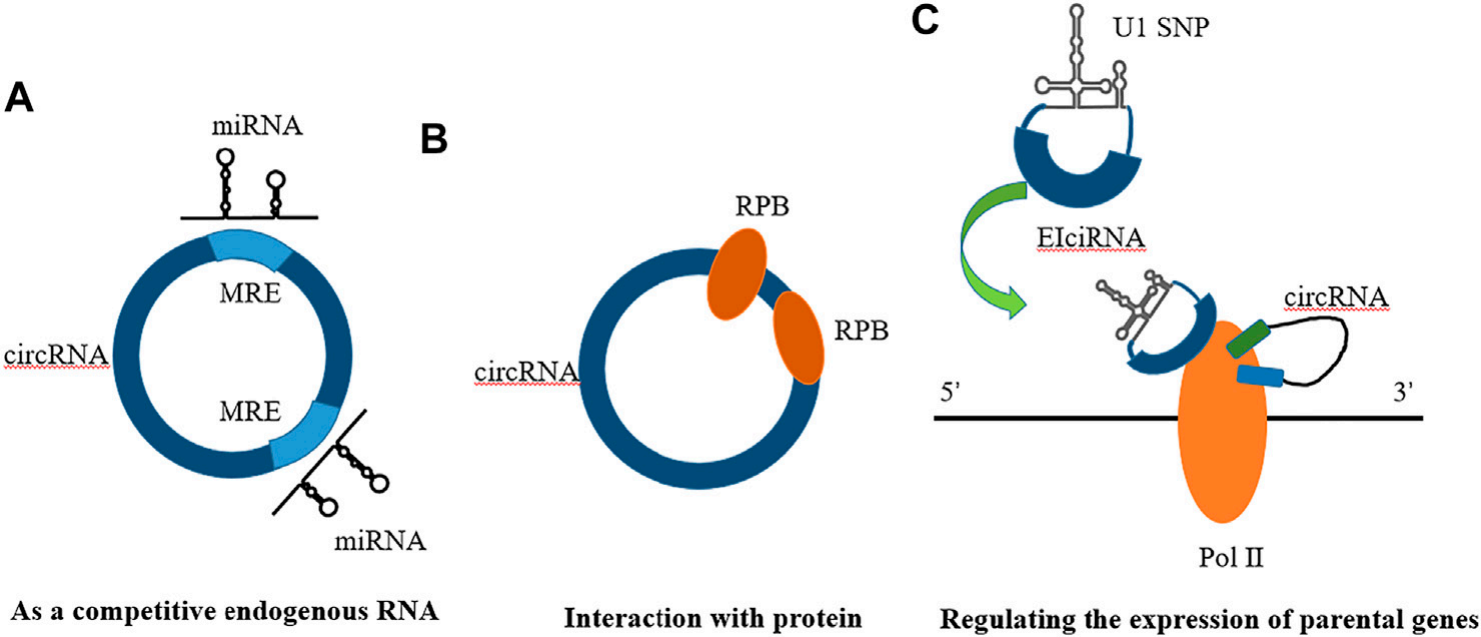
Mechanisms of circRNA action. **(A)** As a compentitive endogenous RNA **(B)** Interaction with protein **(C)** Regulating the expression of parental genes.

Competitive endogenous RNAs (ceRNAs) in organisms can competitively bind to miRNAs, thereby affecting the regulation of gene expression by miRNAs (Figure 4A).

#### Regulating Alternative Splicing or Translational Alternative Splicing (Also Called Alternative Splicing).

Alternative splicing refers to the process of producing different mRNA splice isoforms through different splicing modes (different splice site combinations) from an mRNA. The final protein product shows different or antagonistic functional and structural characteristics compared with unspliced RNA or lead to different phenotypes due to different expression levels in the same cell (Zhang et al., 2016). For example, Zang et al. (2020) found that circRNAs can participate in the regulation of splicing and transcription. Chao et al. (1998) found that the mouse Fmn gene can produce circRNA through “backsplicing”.

**FIGURE 5:**
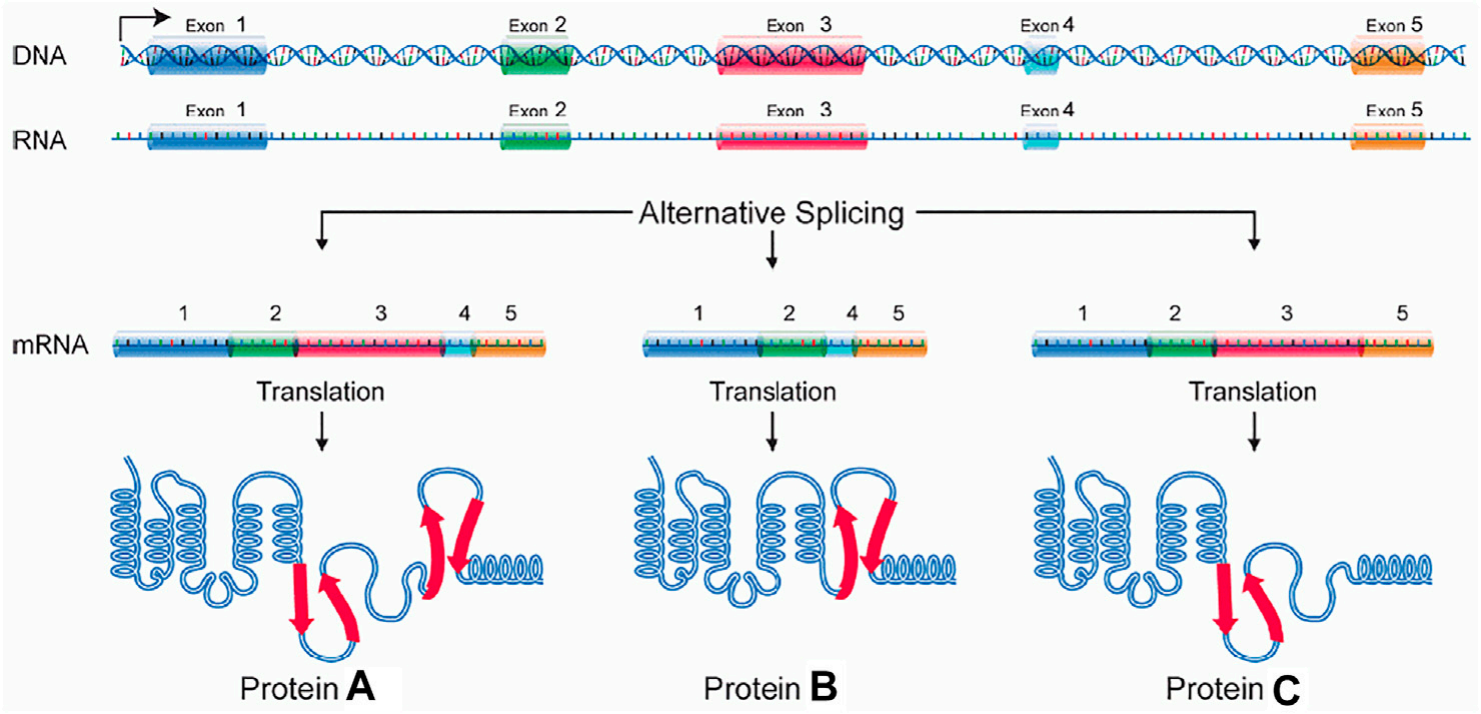
Mechanisms of circRNA action. (http://www.genome.gov/Images/EdKit/bio2j_large.gif).

In previous studies, it was customary to classify circRNAs as noncoding RNAs because they cannot be translated under normal circumstances. However, recent studies have shown that circRNAs can be translated into functional proteins under certain conditions, which will help us refine our understanding of circRNAs and molecular biology (Li et al., 2020). The study of circRNAs is also very helpful in understanding diseases because we can better understand the central role played by translation in different cell types (Figure 5).

#### Regulating the Expression of Parental Genes

Regulating the expression of parental genes is one of the main functions of noncoding RNAs. However, different types of noncoding RNAs have different mechanisms for regulating parental genes. For example, lncRNAs can regulate the expression of parental genes through epigenetic modification of the chromatin state, and noncoding RNAs such as roX1 and roX2 can trans-regulated gene transcription (Kristensen et al., 2019). CircRNAs mainly enhance the expression of their parental genes in cis. On the one hand, circRNAs can facilitate the binding of RNA-binding proteins to each other, affecting the expression of parental gene mRNAs. On the other hand, competitive complementary pairing between introns during circRNA formation can lead to a balanced linear RNA population, thereby influencing the expression of mRNAs and even protein translation (Figure 4C).

#### Interaction With Proteins

Studies have shown that circRNAs, which play a role in RBP assembly or allosteric regulation, can bind RNA-binding proteins (RBPs) and then exert biological functions. For example, circMbl has a strong direct relationship with the MBL protein. Interactions with various subtypes of the MBL protein can promote the production of circMBl through its own exons (Zang et al., 2020). MBL protein expressed in excess leads to a reduction of its own mRNA output by promoting the production of circMBI. In addition, circMBl can also remove excess MBL protein by binding to the MBL protein. Another exon-derived circRNA can interact with the AGO protein and RNA polymerase Ⅱ, thereby acting as a sponge of microRNA and participating in transcriptional regulation. Thus, circRNAs can combine with different protein molecules to enhance interactions between DNA, RNA, and RBPs to promote their biological functions (Figure 4B).

### Research Methods for circRNA

Obtaining massive circRNA data is the basic prerequisite for further analysis and research on the characteristics, functions, and regulatory mechanisms. Currently, there are two main methods for circRNA high-throughput sequencing of specific tissues or cells. One method is to remove linear RNA molecules by nuclease treatment according to the closed loop characteristics of circRNA and then sequence the circRNA molecules after they are specifically enriched. The other method is to sequence RNA directly without nuclease treatment and then screen potential circRNAs through bioinformatics (Hoffmann et al., 2014; Jeck and Sharpless, 2014).

Because circRNA has a stable circular structure and linear RNAs do not have this feature, treatments such as 5′-end exonuclease can degrade most linear RNAs, while circRNAs are not degraded. Thus, circRNA can be initially distinguished from linear RNA. Furthermore, divergent primers and convergent primers used for circRNA amplification can also be designed for cDNA and whole-genome DNA (gDNA). Running a gel can confirm that circRNA can be amplified from cDNA with divergent primers but not from gDNA (Barrett and Salzman, 2016). This outcome is a result of divergent primers promoting formation of the circRNA loop, which can only be formed from single-stranded cDNA; thus, double-stranded gDNA cannot be used to form a loop. There are many validation methods for circRNA functions, such as RIP, which can be used to study the binding of circRNA to protein in cells (Memczak et al., 2013; Li et al., 2015b). Enrichment results of coimmunoprecipitation of AGO2 protein can be used to verify that circRNA binds to target miRNAs. FISH, which can be used to sublocalize circRNA, can interfere with the expression of circRNA because it uses siRNA and antisense oligonucleotides to verify the function of circRNA.

### Regulation of Ruminant Milk Lipids by circRNA

CircRNAs are more stable in nature due to their closed-loop structure, and are generated by reverse splicing and have characteristics of “molecular sponges”. They contain a large number of nucleic acid- and protein-binding sites and can adsorb a large number of regulators, such as miRNAs. Thus, circRNAs can play regulatory roles that exceed the potential of a single miRNA species. As the research on circRNA increases, a large number of findings have shown that circRNAs play important roles in almost every process of milk fat synthesis. There have also been some studies on the roles of circRNAs in ruminants.

Mammary epithelial cell functionality form basis of lactation in ruminants. The number and activity of mammary epithelial cells are closely related to lactation capacity, and these cells play important roles in mammary gland development (Boutinaud et al., 2004). Thus, a better understanding of the molecular mechanisms of mammary epithelial cell development is essential to obtain economic benefits. It has been observed that circRNAs can regulate proliferation and viability of goat mammary epithelial cells through their corresponding miRNAs (Liu et al., 2020a; Zhu et al., 2020). Analogous studies (Liu et al., 2020b) on the effect of circHIPK3 on the proliferation and differentiation of BMECs have been performed with cattle. It was experimentally demonstrated that circHIPK3 promoted proliferation of BMECs, and it was found that the expression of circHIPK3 was significantly reduced in cells treated with prolactin and STAT5 inhibitors. The mechanism of interaction between the STAT5 signaling pathway and circRNAs has been elucidated (Wang et al., 2021), but the mechanism of action between prolactin and circRNAs is not yet clear. Thus in-depth studies would be valuable.

Fatty acid composition plays an essential role in regulating the flavor and quality of dairy products. Chen et al. (2020b) using bovine mammary tissue at different stages reported that these tissues exhibited significantly different circ09863 expression levels. By constructing a circ09863/miR-27a-3p/FASN regulatory network, it was found that overexpression of circ09863 significantly increased TAG and unsaturated fatty acids content, particularly C16:1 and C18:1. Previous findings have demonstrated that FASN expression is correlated with fatty acid content in the mammary glands of ruminants (Roy et al., 2006; Zhu et al., 2014). No study of circRNA and FASN interactions has been performed in sheep. Thus, further studies on circRNA and FASN may provide a breakthrough that researchers can leverage to improve the mechanism of circRNA action in ruminants.

Milk proteins were mentioned previously in the micRNA section, and corresponding studies have also been reported on circRNAs. For example, Zhu et al. (2020) investigated the function and molecular mechanism of circRNA8220 in GMECs and found that there is a circRNA8220/miR-8516/STC2 pathway that promotes the synthesis of ß-casein. Liu et al. (2020a) conducted similar studies that suggested that circ016910 acts as a sponge for miR-574-5p and reduces ß-casein secretion by GMECs.

Overall, circRNAs in ruminants have important regulatory roles that affect the functions of miRNAs. However, there are still relatively few studies on circRNAs in ruminant milk fat regulation, and the specific mechanism of action remains to be studied in detail.

## Competitive Endogenous RNA Mechanisms of Action

The ceRNA mechanism represents a newly discovered gene expression regulatory mechanism (Ebert et al., 2007; Ala et al., 2013). The competitive endogenous RNA mechanism (ceRNA) causes miRNA to bind to mRNA to silence genes, and ceRNA can competitively bind microRNA through response elements (microRNA response elements, MREs) to regulate target gene expression (Smillie et al., 2018). From the biological function of ceRNAs, RNA transcripts dilute the concentration of intracellular free miRNAs by competitively binding miRNAs to coding RNAs, reduce the inhibition of coding RNAs by miRNAs, and then increase the expression of target genes. Overall, ceRNAs have the same miRNA binding sites and ceRNAs are all regulated by miRNAs, and there is a mutual regulatory relationship between ceRNAs, and the trend of expression levels is consistent (Cesana et al., 2011).

There are many types of ceRNAs, such as lncRNAs and pseudogenes. Yue et al. (2019) used high-throughput sequencing data to construct a lncRNA–miRNA–mRNA network based on the ceRNA mechanism theory to perform functional annotation and exploration of lncRNA ceRNAs. As a result, they found a scale-free feature of the network that has an effect on the development of bovine skeletal muscle. With high functional specificity, this study provided insights into the function and mechanism of lncRNA-mediated ceRNA in bovine skeletal muscle development. As another example, Yu et al. (2017) conducted experiments on mRNA, miRNA, and lncRNA in breast mammary at two time points (early and mature) during lactation in goats. The results showed that ceRNA (lncRNA-mRNA) may be involved in the lactation process, and plays an important role in different stages of lactation. Compared with reports on humans, especially related to cancer, reports dealing with ruminants are quite few. For example, researchers at Beth Israel Deaconess Medical Center (BIDMC) found that the pseudogene BRAF acts as a ceRNA and induces an aggressive lymphoma-like disease. This study showed that pseudogenes may play an important role in various diseases.

Recently, circRNAs have also been suggested to constitute a subpopulation of ceRNAs that can act similar to a sponge and absorb miRNAs by base complementary pairing, thereby regulating the expression of miRNA target genes. For example, Zhang et al. (2018) found in the prereceptive endometrium of dairy goats that ciR3175 can reduce the level of miR-182 by acting as a sponge of miRNA, and miR-182 can downregulate TES in endometrial epithelial cells (EECs) through the target site, as predicted, *in vitro*. In this way, ciR3175 acts as a ceRNA that isolates miR-182, thereby protecting TES transcripts from miR-182-mediated EEC inhibition *in vitro*.

RNA transcripts regulate each other through their shared MREs. The greater the number of shared MREs is, the greater the degree of MRE communication or coregulation. Thus, ceRNAs can form a large-scale transcriptional regulatory network to promote interactions among lncRNAs, miRNAs, and circRNAs through MREs and thus, constitute a complete and complex ceRNA molecular regulatory network by competing for MREs.

**FIGURE 6:**
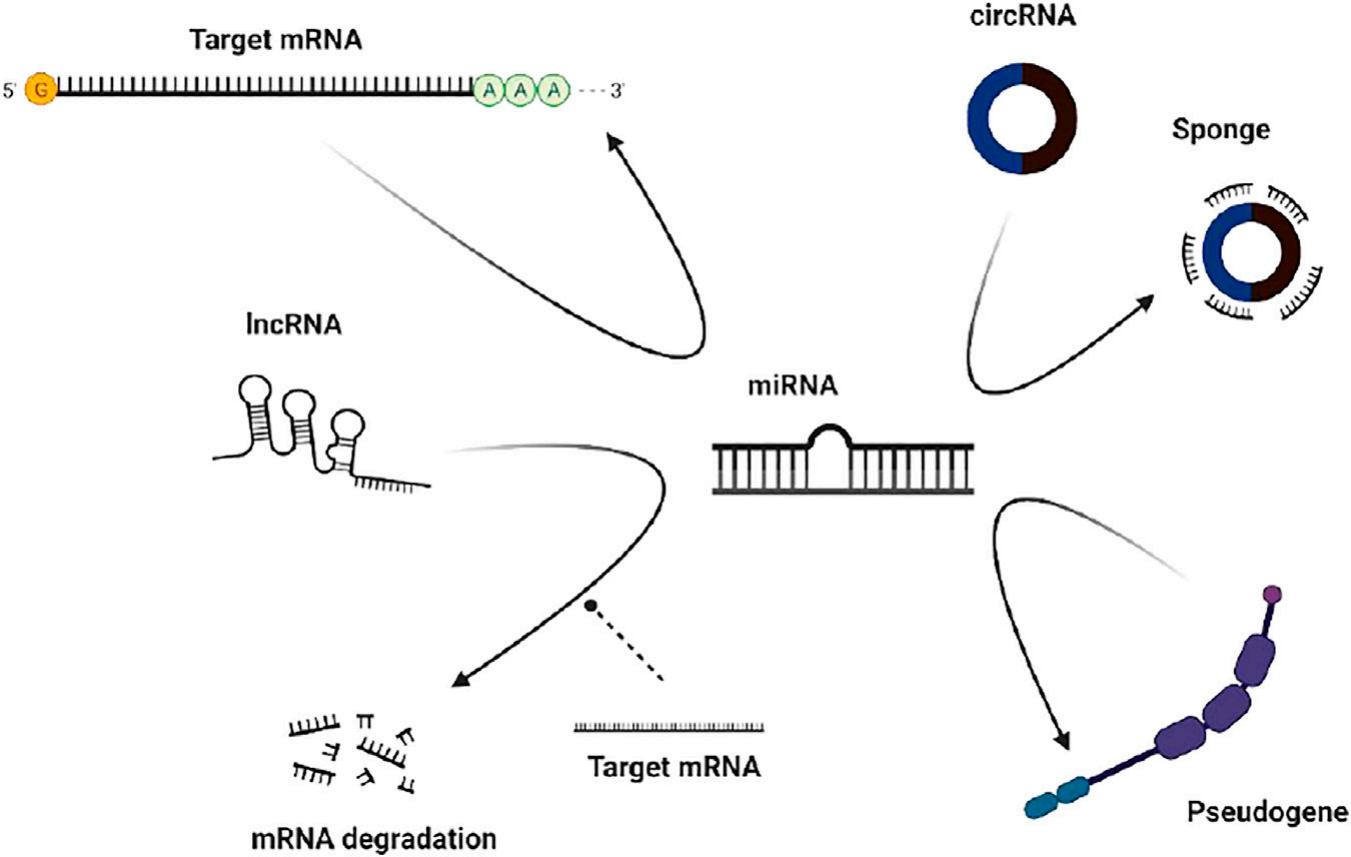
ceRNA mechanisms.

Compared with the microRNA regulatory network, the ceRNA network, as a novel gene expression regulation model, is more sophisticated and complex and involves more RNA molecules (Figure 6). The interaction between lncRNAs and miRNAs plays an important role in many diseases. There are more lncRNAs than mRNAs, and they are more cell-specific than mRNAs. In addition to directly participating in the regulation of the occurrence and development of diseases, lncRNAs can also interact with other macromolecular substances, such as DNA, RNA, and proteins, to jointly participate in the regulation of the occurrence and development of diseases. In addition to protein scaffolds and gene interaction mechanisms, ceRNAs have been proposed to be participants in novel LncRNA mechanisms.

CircRNAs with miRNA-binding sites can function as ceRNAs. CircRNAs compete with other RNAs through RNA-binding proteins (RBPs) or miRNAs. The circRNA sequence is significantly enriched in conserved nucleotides and can resist exonucleases. Degradation of the very abundant endogenous circRNA molecules is an effective method of miRNA “sponging” to enrich the regulatory functions of gene expression.

## Discussion

**FIGURE 7:**
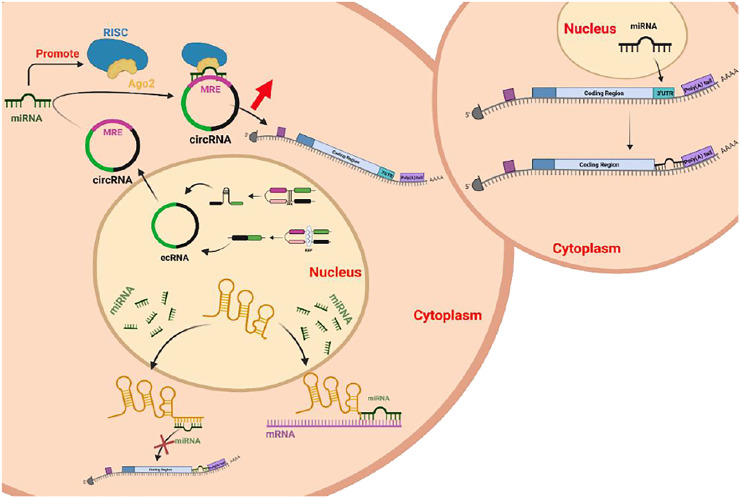
MiRNAs under normal and competing endogenous conditions.

As an increasing number of researchers pay attention to and work in the field of ncRNA research, our understanding of ncRNAs is becoming increasingly more profound. Not only do ncRNAs perform their biological functions in organisms through a variety of mechanisms, but their dysfunction is related to the occurrence and development of a variety of diseases (Mattick and Makunin, 2006; Mercer et al., 2009; Matsui and Corey, 2017; Anastasiadou et al., 2018; Slack and Chinnaiyan, 2019). After the ceRNA mechanism was proposed, noncoding RNA have been associated with this process. Using miRNAs as bases, circRNAs and lncRNAs bind to microRNA response elements (MREs) to form signaling pathways or axes that affect gene expression. Compared with the miRNA regulatory network, the ceRNA mechanism that leads to increased circRNA or lncRNA levels is more sophisticated and complex and involves more RNA molecules (Figure 7). This novel gene expression regulation mode is expected to produce a new research hotpot. The study of the ceRNA mechanism is gradually expanding, and more problems that have puzzled humans for a long time are being solved, including those related to cancer treatment, gene-editing technology improvement, and plague prevention. Additional new technologies have been developed that facilitate research and also enrich people’s lives.

Of course, while we are constantly studying new mechanisms, we must not forget to continue to explore miRNAs, lncRNAs, and circRNAs. Due to the diversity and complexity of the mechanisms of ncRNAs, the current understanding of their functions encompasses only the tip of the iceberg and has not been fully utilized; the potential of ncRNAs has not yet been fully tapped.

Although studies based on established methods such as CRISPR/Cas9 and other technologies have proven that miRNAs can improve milk fat percentage, and these findings have been applied to ruminant research through *in vitro* experiments, the difficulty in operating and the high cost render the “use of miRNAs to improve milk fat in ruminants” challenging. Thus, we still need to develop new technologies to translate existing results into production to truly bring economic benefits to the breeding industry and provide consumers with better milk products. Furthermore, many new technical methods have recently emerged including high-throughput miRNA microarrays, but there are few relevant research reports. In the authors’ opinion, using the latest miRNA technology as well as in-depth studies of the relationship between diseases will be very helpful in understanding gene expression regulatory networks in higher eukaryotes. Similarly, on the basis of research, if the relevant results can be invested in practical applications, great progress will be made in multiple fields such as the application of miRNA-based blood or urine tests to protect cancer patients from the pain of invasive exploration.

Currently, a breakthrough has been made in the mechanism, classification, and identification of lncRNAs and circRNAs. However, there are few reports on their transcriptional regulation, functional mechanisms, and signal transduction, and experimental validation strategies still need to be further explored. However, it can be clarified that, in the regulation of milk fat metabolism, lncRNAs and circRNAs act indirectly on lipogenic genes through miRNAs and have a positive effect on the expression of genes, which is of great biological significance in regulating the process of milk fat metabolism.

Existing studies on the functions of circRNA in regulating milk fat metabolism in ruminants mainly focus on circRNAs acting as sponges of miRNAs and thus regulating downstream target genes or pathways. However, other functions have not been studied in depth, such as the translational function of circRNAs and their translation products. The authors believe that the relationship between circRNA and milk fat metabolism should be studied next and that the further elucidation of the interaction mechanisms will help in understanding the mutual regulatory relationship between circRNA and milk fat. These studies will also contribute to the development of biomarkers and treatment targets for the improvement of milk fat.

In contrast to that of circRNAs, the understanding of most ruminant lncRNAs remains elusive thus far. The authors believe that the reason for this situation is mainly that lncRNAs are highly specific, and the role of lncRNAs may be observed only under specific conditions. Most lncRNAs are poorly conserved between species, and the research results of many existing species are not the same as those obtained for ruminants. LncRNAs are heterogeneous and able to bind to sequences of regulatory DNA, which makes it difficult to assess their specific functions. Thus, more effective methods and techniques need to be established to more deeply and rapidly explore the biological functions of lncRNAs in ruminants and find similarities and differences in lncRNAs between different species and among ruminants. Then, a more complete exploration and understanding of the substantial roles of lncRNAs in the regulation of milk fat metabolism in ruminants and the mechanisms by which they play these roles can be achieved.

We can affirm that there are still gaps in the research of miRNA, lncRNA, circRNA, and ceRNA mechanisms in the regulation of milk fat. Additionally, the specific mechanism of action remains to be more deeply studied and explored to fully comprehend milk fat metabolism mechanisms in ruminant livestock. Furthermore, milk fat content is not only epigenetically regulated by ncRNAs but also greatly affected by feeding and management levels, body hormone secretion levels, and dietary nutrient quality. Thus, combining these factors with ncRNAs to determine the inevitable link among them will be important for making genuine improvements in milk fat production. Clearly, there is no doubt that the newly discovered circRNAs must not be the last members in the family of ncRNA to be studied; there will be more biological mysteries waiting for us to explore.
